# The Efficacy and Safety of Hypofractionated Radiation Therapy With Tomotherapy for Advanced or Recurrent Hepatocellular Carcinoma

**DOI:** 10.3389/fonc.2021.559112

**Published:** 2021-05-31

**Authors:** Jie Shen, Jing Yan, Sihui Zhu, Weiwei Kong, Zhengyun Zou, Juan Liu, Shuangshuang Li, Baorui Liu

**Affiliations:** ^1^ Comprehensive Cancer Centre of Nanjing Drum Tower Hospital, Clinical College of Nanjing Medical University, Nanjing, China; ^2^ Comprehensive Cancer Centre of Drum Tower Hospital, Medical School of Nanjing University, Clinical Cancer Institute of Nanjing University, Nanjing, China

**Keywords:** tomotherapy, hepatocellular carcinoma, immunotherapy, PD-1, hypofractionated

## Abstract

The effects of radiotherapy on hepatocellular carcinoma (HCC) still remain to be further proved. The dose of radiotherapy is generally 2Gy*25f. In the current study, we prospectively investigated the clinical outcomes of advanced or recurrent HCC patients who received hypofractionated radiotherapy at a dose of 5Gy*10f with tomotherapy. A study involving hypofractionated radiotherapy (5Gy*10f) based on TOMO was conducted in HCC patients with Child-Pugh grade A or B who were unsuitable candidates for resection or radiofrequency ablation or with residual disease after transarterial chemoembolization (TACE). The prescription dose was 50 grays in 10 fractions. From Sep. 2016 and Dec. 2017, 65 patents were evaluated with a median follow-up of 24 months (range: 7–41 months). 10 patients were treatment-naïve (failure to undergo surgery or intervention due to the presence of a portal or portal branch tumor thrombus), 15 patients were treated for residual HCC after TACE as salvage therapy, and 40 cases were treated for recurrent HCC. The median overalls survival (OS) of these patients was 18 months. Among them, 27 patients classified as BCLC stage B had a median OS of 22 months. Moreover, 28 patients classified as BCLC stage C had a median OS of 14 months. None of the patients experienced recurrence in the area of radiotherapy. The local control rate of primary tumor at 3 months, 6 months, 1 year and 2 years was 100%. The 3-month survival rate was 100%, the 6-month survival rate was 100%, the 1-year survival rate was 75.4%, and the 2-year survival rate was 43%. In addition, 14 patients had the opportunity to continue the treatment of PD-1 antibody after the disease progression, and their prognosis was not surprisingly better compared with the patients who did not receive PD-1 antibody treatment (NR *vs*. 15 months, P=0.04). No serious side effect was found in all patients during and after radiotherapy. Hypofractionated radiotherapy (5Gy*10f) based on TOMO achieved high local control rate and OS with tolerable toxicities for HCC patients. TOMO therapy could be used to effectively against HCC in treatment-naive, intrahepatic failure, residual disease, and recurrent settings.

## Introduction

Curative treatments for patients with early-stage hepatocellular carcinoma (HCC) include resection, liver transplantation (LT) and percutaneous ablation therapy. However, only <30% of patients are eligible for these treatments. In addition, HCC patients are not always suitable for these treatments, presumably because of poor liver function and/or tumor location. In such situations, the patients usually receive transarterial chemoembolization (TACE). However, the local control after TACE remains unsatisfactory (1, 2).

Stereotactic body radiotherapy (SBRT) is a high-precision, conformal, external-beam radiation technique that ablates the target at extracranial sites using hypofractionated, high-dose radiation while sparing surrounding normal tissues. Currently, SBRT is considered a treatment option for patients with medically operable, early-stage, non-small cell lung cancer (3). Findings from retrospective studies of patients with small HCC who receive SBRT have indicated high local control rates from 99% to 100%, showing minimal blood vessel and bile duct toxicities. In addition, phase 2 studies consisting of HCC patients with large or locally advanced disease have also demonstrated high efficacy and safety (4, 5). However, the effect of SBRT remains unclear in those with intrahepatic failure, residual disease, and recurrent settings.

In the current study, we prospectively investigated the clinical outcomes of hypofractionated radiotherapy (5Gy*10f) with TOMO for patients with Child-Pugh grade A or B who were unsuitable candidates for resection or radiofrequency ablation or with residual disease after TACE.

## Materials and Methods

### Patient Characteristics

A total of 65 patients unsuitable for resection or radiofrequency ablation and those with recurrent or residual disease after TACE were recruited in this retrospective study. The patients were treated with a hypofractioned dose-escalated regimen between Sep. 2016 and Dec. 2017 in the Comprehensive Cancer Center of Drum Tower Hospital of Nanjing University. The medical records of all patients were carefully reviewed. Inclusion criteria were set as follows (1): diagnosed with HCC by pathology (2); unsuitable candidates for resection (3); recurrent and residual diagnostic criteria: in HCC, after TACE in the CT/MR examination, it was found that the tumor had subsequent reappearance or the enhancement of the tumor was defined as recurrence, or decreased but remained defined as residual (4); a history of liver cirrhosis derived from hepatitis B, and hepatitis B virus DNA <500 copies/mL (5); class A and B liver function by Child-Pugh scoring (6); PS 0-1 (7); a volume of normal liver tissue > 700 mL (8); white blood cell (WBC) count>3.0*10^^9^, hemoglobin (HB)> 90 g/L, platelet (PLT) count> 70*10^^9^. During treatment, targeted drugs, such as sorafenib, and immunotherapy, such as GM-CSF, could be combined when necessary. The median age of the 65 patients was 58.63 years (range: 38–83), and 89% of them were males. Cisplatin, adriamycin, and/or mitomycin C were administered during TACE. Patient characteristics were presented in **Table 1**.

**Table 1:** Patient characteristics.

Characteristic																																		No. of patients (%)
Age																																		58.63	
Sex																																			
men																																		58 (89%)	
women																																		7 (11%)	
ECOG PS																																			
0																																		31 (47%)	
1-2																																		34 (53)	
Greatest tumor dimension																																			
Treatment-naive																																		10 (15%)	
Intrahepatic Residual																																		15 (23%)	
Recurrent																																		40 (62%)	
UICC-TNM stage at treatment																																			
T1																																		0	
T2																																		12 (18%)	
T3																																		25 (39%)	
T4																																		28 (43%)	
BCLC stage at treatment																																			
A																																		10 (15%)	
B																																		27 (42%)	
C																																		28 (43%)	
Tumor site Liver Lung Soft mass in iliac bone LN PVTT Bone Child-Pugh score																																		37 (57%) 10 (15%) 5 (8%) 9 (14%) 2 (3%) 2 (3%)	
A																																		51 (78%)	
B																																		14 (22%)	
C																																		0	
Type of chronic hepatitis																																			
HBV																																		60 (92%)	
HCV																																		0	
Alcoholic																																		5 (8%)	
Other																																		0	
Tumor marker																																			
AFP: Median [range], ng/mL																																			
0-10																																		1 (2%)	
>10																																		64 (98%)	
Cytokines (GM-CSF/IL-2) YES																																		41 (63%)	
NO																																		24 (37%)	
Sorafenib																																			
YES																																		17 (22%)	
NO																																		48 (78%)	
PD-1 after PD																																			
YES																																		14 (22%)	
No																																		51 (78%)	

ECOG, Eastern Cooperative Oncology Group; PS, performance status; UICC, Union for Cancer Control; BCLC, Barcelona Clinic Liver Cancer; LN, lymph node; PVTT, portal vein tumor thrombosis; HBV, hepatitis B virus; HCV, hepatitis C virus; PD-1, programmed cell death-1; PD, progressive disease.

### Radiation Therapy

The tumor area was identified and outlined using a plain CT scan image as the basis to fuse CT/MR enhanced images. To be safe, some necessary parameters and indicators were explored. For example, the dose of the average liver was controlled below 22 Gy, and the volume was guaranteed to be above 700 mL (the volume of a normal liver was above 700mL). Specially protected liver was intentionally circled, the average dose was controlled below 8 Gy, and the volume was guaranteed to be above 400 mL. A plan was carried out using the helical tomotherapy system (Accuray Incorporated, Sunnyvale, CA, USA). Target and critical structures (spinal cord, lung, heart, esophagus, remaining healthy liver, stomach, intestine, and kidneys) were contoured. The GTV (gross target volume) was defined as a tumor visible on the CT/MR scan. The GTV was expanded by 10-12 mm to form the PTV (plan target volume), which was expanded by 5 mm to form the PGTV (plan gross tumor volume). All patients were treated with a total dose of PGTV 50 Gy/10f and PTV 30 Gy/10f. Plans were devised so that the prescribed dose was the isodose line encompassing >96% of the PTV. No more than 3% of the PTV was to receive <94% of the prescribed dose.

### Alpha-Fetoprotein (AFP)

An index AFP level was recorded before radiotherapy. AFP levels were also recorded at 3 months, 6 months, 1 year and 2 years after treatment.

### Toxicity

Toxicity induced by SBRT was scored according to the NCI Common Terminology Criteria for Adverse Events (CTCAE) version 4.03. Radiation-induced liver disease (RILD) was defined as an anicteric elevation in alkaline phosphatase of at least 2-folds of the upper normal level (classic RILD) or elevated transaminases of at least 5-folds (non-classic RILD), without progressive disease (PD) and the development of nonmalignant ascites. Acute toxicities were defined as those occurring within 90 days of SBRT. Late toxicities were defined as those occurring afterwards.

### Statistical Analysis

Survival rates were calculated from the date of SBRT. Kaplan-Meier survival analysis was used to estimate overall survival (OS). All statistical analyses were conducted by Graphpad version 8.0. *P* < 0.05 was considered as statistically significant.

## Results

**Figure 1:**
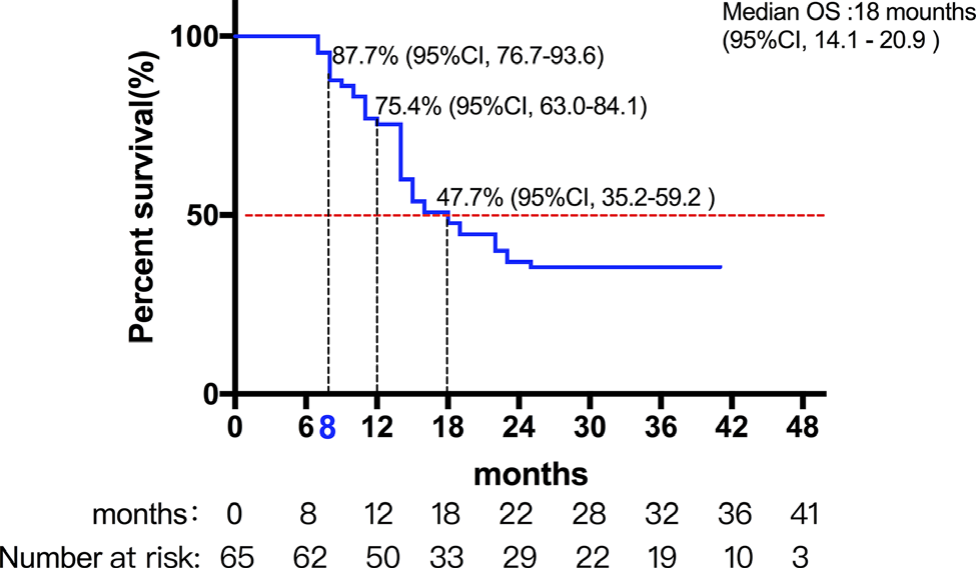
The median OS of these patients.

A total of 72 patients were evaluated between 2016 and 2017. Among them, seven patients were lost during follow-up, and 65 patients were included in the final analysis. **Table 1** lists the detailed pretreatment characteristics of the evaluable patients. The median follow-up time was 24 months (range: 7–41 months). The median OS of all the patients were 18 months (95%CI, 14.1-20.9) (**Figure 1**. Among all evaluable patients, 10 were treatment-naive for HCC (failure to undergo surgery or intervention due to the presence of a portal or portal branch tumor thrombus); and 15 patients received treatment for newly diagnosed intrahepatic failure or residual after TACE, and another 40 patients were recurrent HCC. TACE was given as scheduled treatment to 15 patients who underwent SBRT. The median interval between TACE and SBRT was approximately 1.0-2.0 months. There were 28 patients who were classified as BCLC stage C, and the number of patients classified as BCLC stage B was 27. Among the 28 patients classified as BCLC stage C, 13 patients received radiotherapy in combination with sorafenib. In all of 65 patients, 41 patients received radiotherapy in combination with cytokines (such as GM-CSF or IL-2). Moreover, 14 patients had the opportunity to continue the treatment of PD-1 antibody after PD.

### Survival Rate

**Figure 2:**
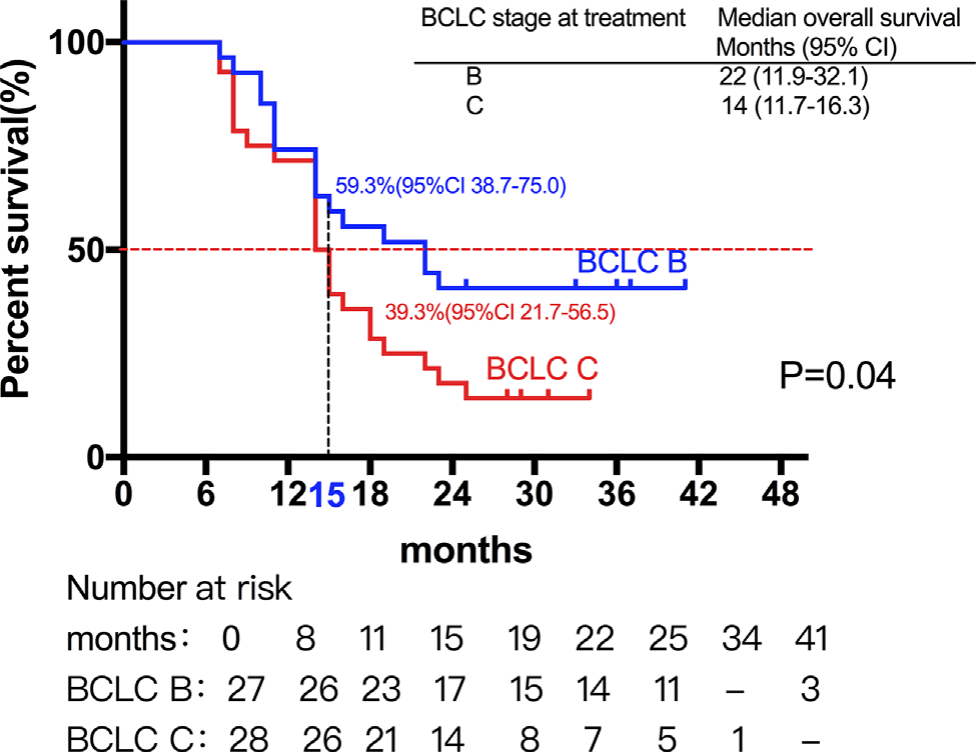
Median OS of patients with different stage. A total of 27 patients classified as BCLC stage B had a median OS of 22 months, while 28 patients classified as BCLC stage C had a median OS of 14 months.

**Figure 3:**
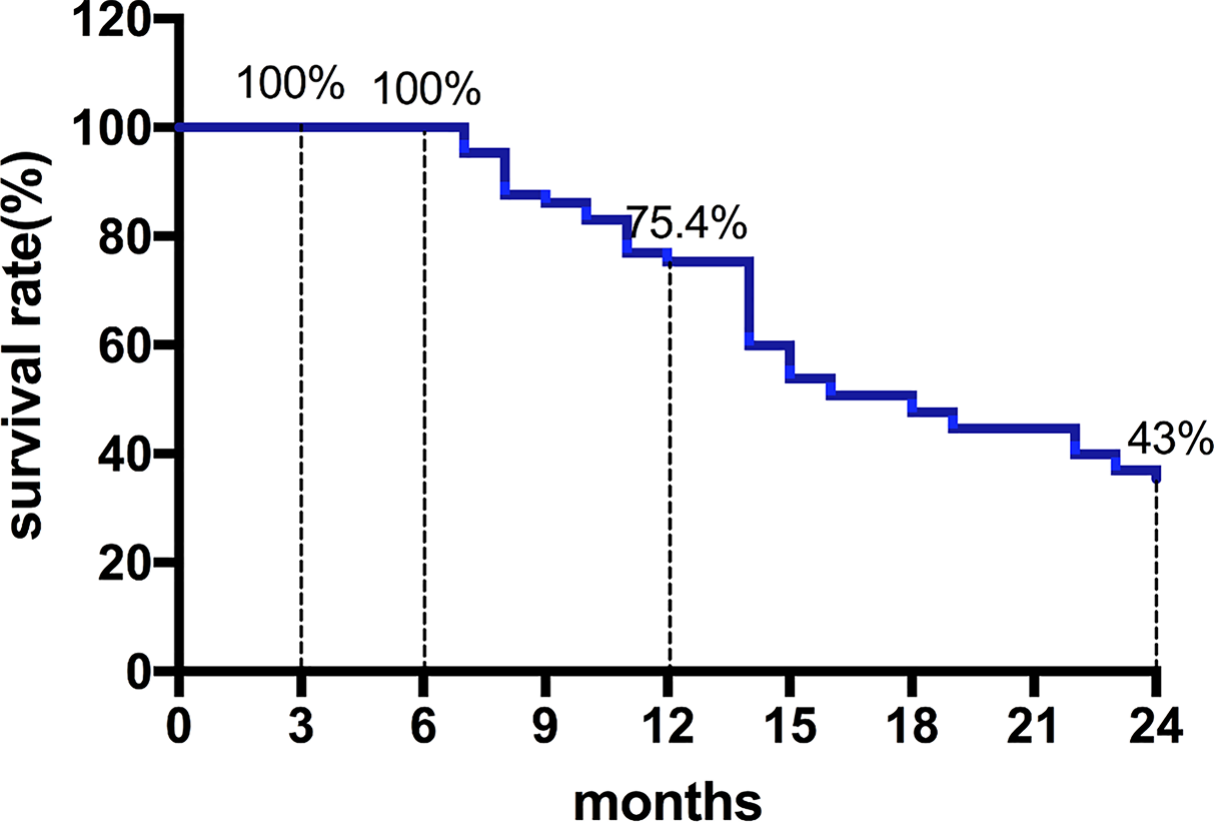
The survival rate curve. The 3-month survival rate was 100% (65/65), the 6-month survival rate was 100% (65/65), the 1-year survival rate was 75.4% (50/65), and the 2-year survival rate was 43% (28/65).

Among them, 27 patients classified as BCLC stage B had a median OS of 22 months (95%CI, 11.9-32.1). Moreover, 28 patients classified as BCLC stage C had a median OS of 14 months (95%CI, 11.7-16.3) (**Figures 2** and **3**) shows that the 3-month survival rate was 100% (65/65), the 6-month survival rate was 100% (65/65), the 1-year survival rate was 75.4% (50/65), and the 2-year survival rate was 43% (28/65).

### Disease Control Rate (DCR)

**Figure 4:**
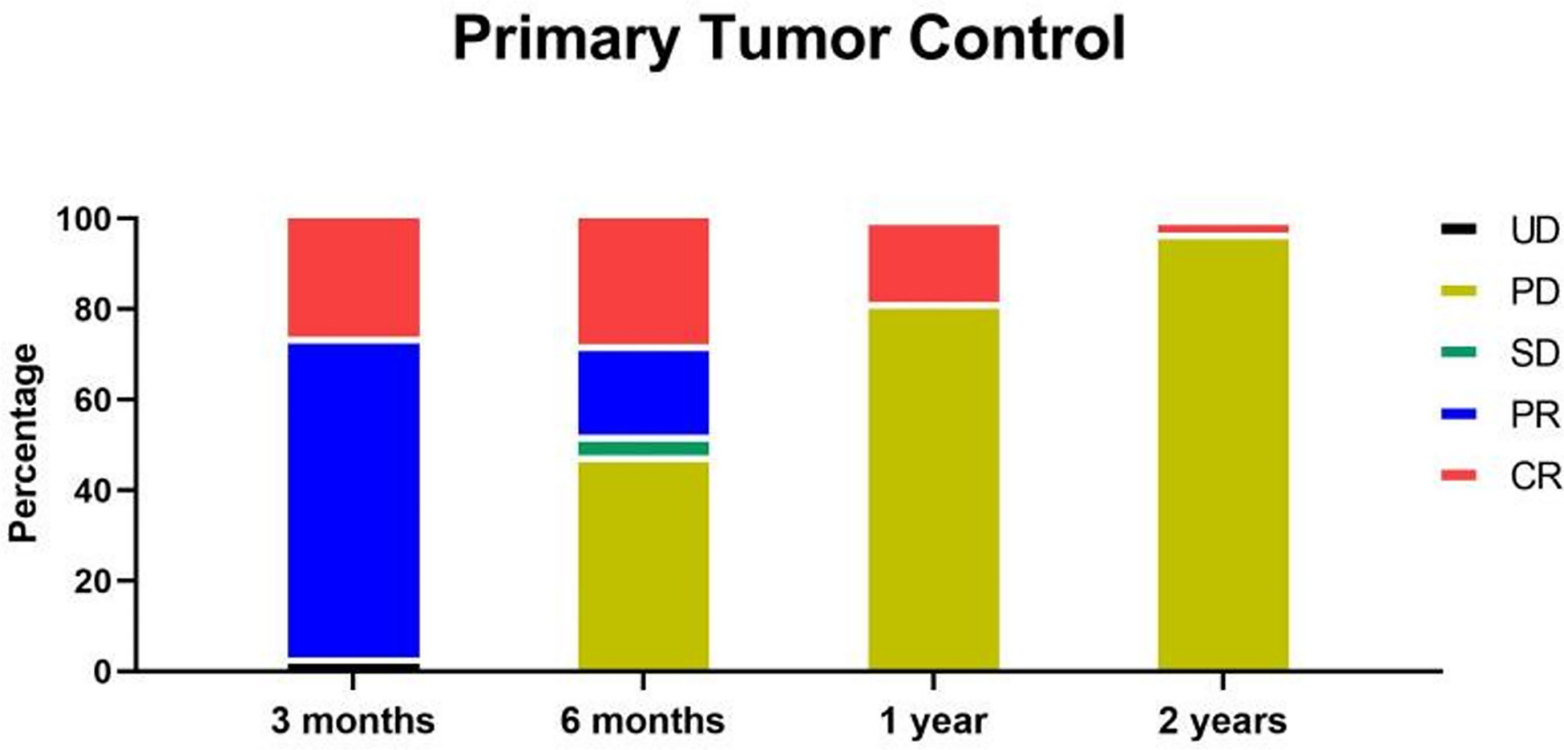
At 3 months after radiotherapy, 18 patients (27.7%) reached CR, 45 patients (69.2%) reached PR, and two patients (3.1%) showed UD. At 6 months after radiotherapy, 18 patients (27.7%) were still CR, 13 patients (20.0%) were still PR, three patients (4.6%) were stable (SD), and 31 patients (47.7%) were progress (PD), including two patients with UD. At 1 year after radiotherapy, 12 patients (18.5%) were still CR, and the rest patients were all PD. At 2 years after radiotherapy, two patients (3.1%) were still CR, and the rest patients were all PD.

At 3 months after radiotherapy, 18 patients (27.7%) reached complete recovery (CR), 45 patients (69.2%) reached partial recovery (PR), and two patients (3.1%) showed unmeasurable tumor (UD). At 6 months after radiotherapy, 18 patients (27.7%) were still CR, 13 patients (20.0%) were still PR, three patients (4.6%) were stable (SD), and 31 patients (47.7%) had progressive disease (PD), including two patients with UD. The newly developed masses or enlarged tumors were all not the primary tumors covered by radiotherapy (**Figure 4**).

At 1 year after radiotherapy, 12 patients (18.5%) were still CR, and the rest patients were all PD. At 2 years after radiotherapy, two patients (3.1%) were still CR, and the rest patients were all PD (**Figure 4**). Fortunately, at 2 years follow up, all the tumors covered by radiotherapy were well controlled, and no recurrence was observed. Moreover, all the PD belonged to newly developed masses or enlarged tumors not covered by radiotherapy. Therefore, the primary tumor local control of radiotherapy (DCR for tumor within the radiotherapy) was 100% in at least 2 years by the dose of 5Gy*10f. The total DCR was 100% for 3 months, 52.3% for 6 months, 18.5% for 1 year, and 3.1% for 2 years.

### AFP

At 3 months after radiotherapy, the AFP level of 14 patients (21.5%) returned to the normal level, the AFP level of 50 patients (77.0%) was decreased but not to the normal level, and one patient (1.5%) showed AFP negative. No patient showed increased AFP level. At 6 months after radiotherapy, 17 patients (26.2%) were still within the normal AFP range, while 32 patients (49.2%) showed increased AFP level, indicating systemic progression. At 1 year after radiotherapy, 11 patients (22.0%) were still within the normal range of AFP, while the rest patients (76.0%) all exhibited increased AFP level except for one negative. We found that one patient had tumor with CR, while the AFP level was increased, indicating the possible progression in the future. At 2 years after radiotherapy, only two patients were still CR, and the rest patients showed increased AFP level.

### Safety Analysis

For the radiotherapy safety analysis, the overall tolerance of patients was quite good (**Table 2**). Transaminase elevation graded 1-2 was found in nine patients (13.8%), and only one patient experienced elevation graded 3-4. Even though, the transaminase level of this patient became normal soon by suitable treatment for liver function protection, and the rest of the patients had no obvious abnormalities. Bilirubin elevation graded 1-2 was detected in three patients (4.6%), and the others all had no obvious abnormalities. Moreover, the albumin level was decreased by 1-2 grades in seven patients (10.8%), and the others all had no obvious abnormalities. PLT (platelet) level was decreased in 22 patients (33.8%) with 1-2 grades, and no obvious changes were found in the others. Alkaline phosphatase level was increased by 1-2 grades in five patients (7.7%), and no obvious changes were found in the others. In addition, three patients (4.6%) had a decrease of 2 points in PS (performance status), while there were no significant changes in the others. Besides, 15 (23.1%) patients exhibited mild anorexia during radiotherapy, while no severe adverse reactions, such as gastrointestinal bleeding, were found.

**Table 2:** Toxicities after hypofractionated radiotherapy (5Gy*10f) based on TOMO.

Toxicity parameters					Toxicity: No. of patients (%)			
Laboratory tests							Grade 1-2	Grade 3-4
Elevated transaminases							9 (13.8%)	1(1.5%)
Hyperbilirubinemia							3 (4.6%)	0
Hypoalbuminemia							7 (10.8%)	0	
Decreased platelet counts							22 (33.8%)	0
Elevated alkaline phosphatase							5 (7.7%)	0
Worsening of PS by 2 points							3 (4.6%)	0

### Combination Treatment Analysis

**Figure 5:**
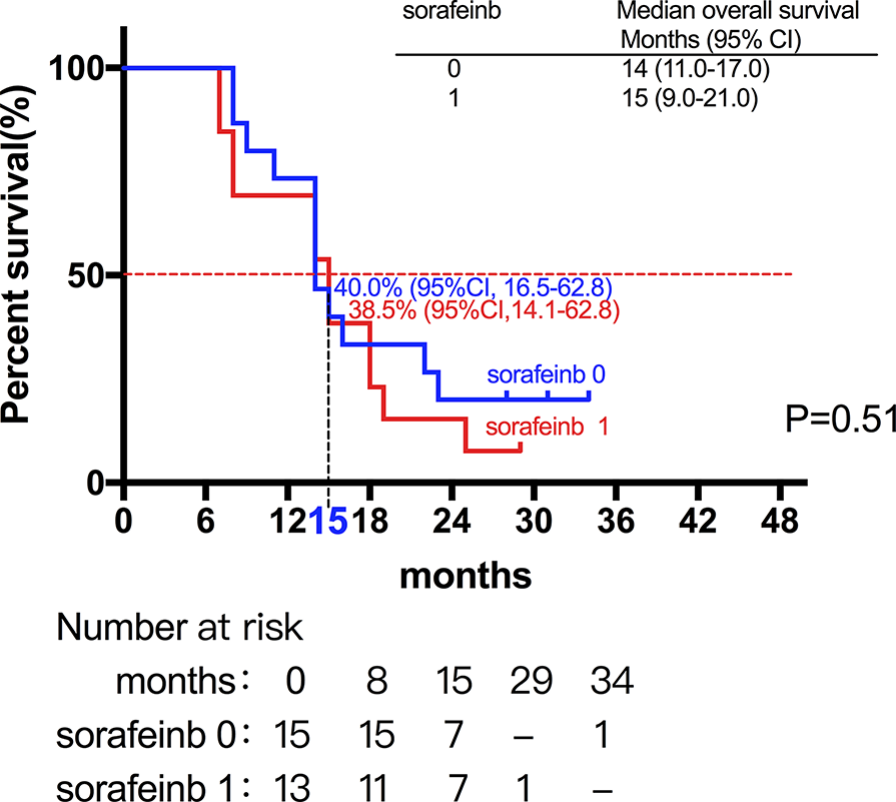
The combination of sorafenib (n=13) and radiotherapy only (n=15) had little effect on the prognosis (15 *vs*. 14 months, P=0.51).

**Figure 6:**
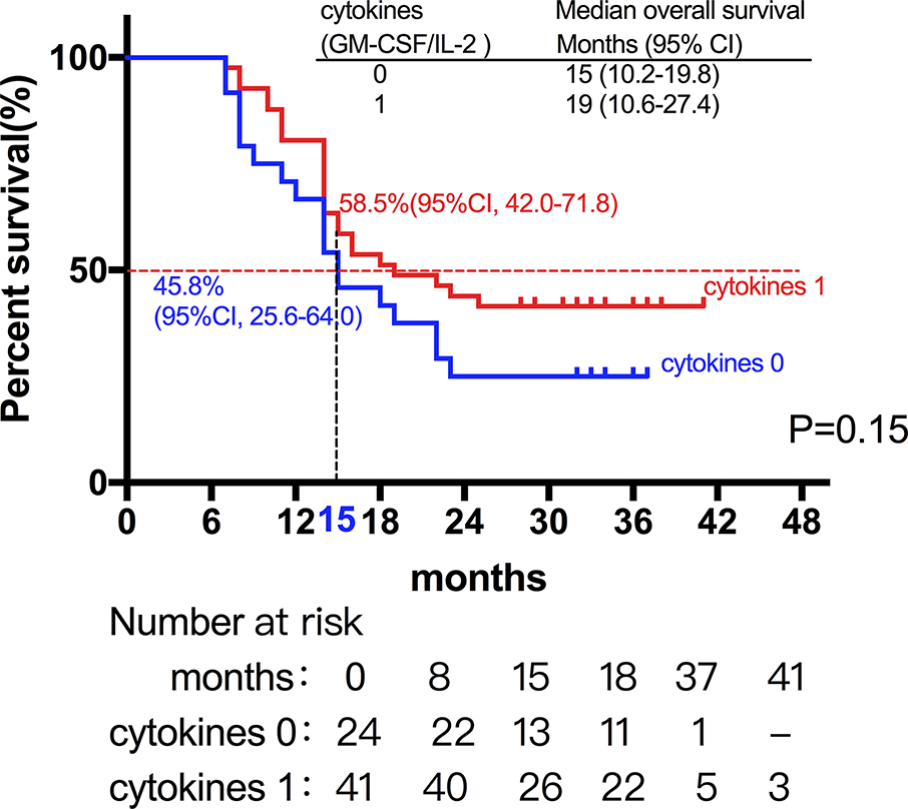
The prognosis of patients receiving cytokine treatment (GM-CSF/IL-2) in combination with radiotherapy was better than that of patients treated with radiotherapy alone, while there was no statistical difference (19 *vs*. 15 months, P=0.15).

**Figure 7:**
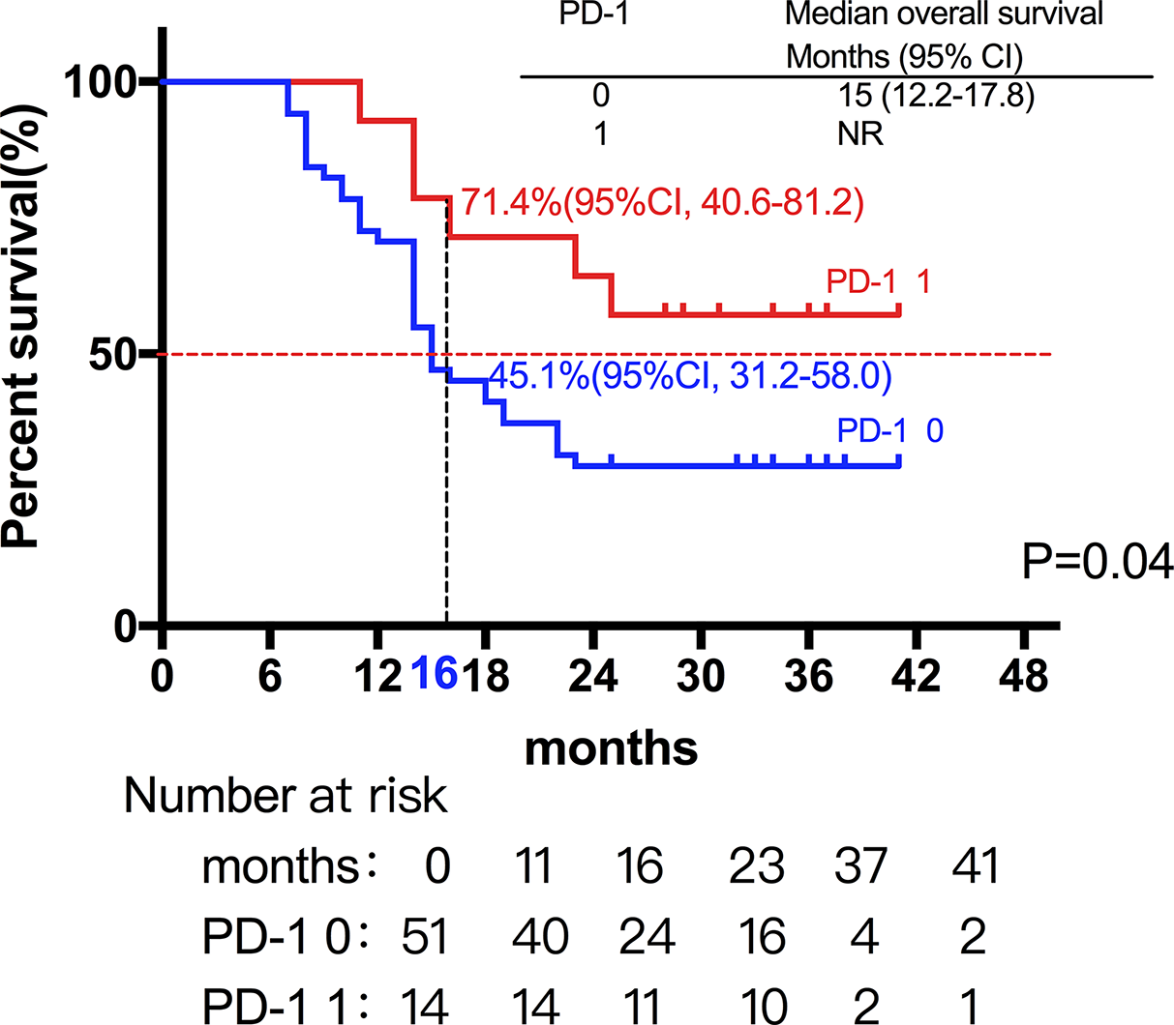
A total of 14 patients had the opportunity to continue the treatment of PD-1 antibody after PD, and the prognosis was not surprisingly better compared with the patients without PD-1 treatment (NR *vs*. 15 months, P=0.04).

Among the 28 patients with grade C HCC, 13 patients received radiotherapy in combination with sorafenib, while the combination of sorafenib (n=13) and radiotherapy only (n=15) had little effect on the prognosis (15 *vs*. 14 months, P=0.51, **Figure 5**). Of all these patients, 41 received the combination therapy (GM-CSF/IL-2). We found that the prognosis of patients receiving combination therapy was better than that of patients treated with radiotherapy alone, while there was no statistical difference (19 *vs*. 15 months, P=0.15, **Figure 6**). Moreover, 14 patients had the opportunity to continue the treatment of PD-1 antibody after PD, and the prognosis was obviously better compared with the patients without PD-1 treatment (NR *vs*. 15 months, P=0.04, **Figure 7**). To get further insight of the role of immunotherapy, multivariable analyses of survival time in all the patients were carried out. As shown in **Table 3**, BCLC stage definitely had relationship with OS. Patients with PD-1 treatment had lower mortality risk compared with those without PD-1 therapy (HR [hazard ratio] of death = 0.32, 95% CI = 0.12 to 0.81, P =0.017).

**Table 3:** Cox regression analysis of overall survival of patients receiving radiotherapy.

Variable	No. of patients	Overall survival		Univariate		Multivariate	
		Month (95%CI)	P value	HR (95%CI)	P value	HR (95%CI)	P value
BCLC			0.003				
A	10	33.0 (26.8-39.2)		1.00 (referent)		1.00 (referent)	
B	27	25.1 (19.9-30.3)		3.85 (0.88-16.74)	0.073	5.56 (1.26-24.53)	0.024
C	28	16.9 (13.7-20.0)		7.08 (1.66-30.17)	0.008	9.93 (2.19-45.09)	0.003
PD-1			0.041				
No	51	21.6 (18.0-25.2)		1.00 (referent)		1.00 (referent)	
Yes	14	30.8 (20.3-26.8)		0.43 (0.18-1.03)	0.057	0.32 (0.12-0.81)	0.017
sorafeinb			0.041				
No	48	26.3 (22.3-30.2)		1.00 (referent)		1.00 (referent)	
Yes	17	15.2 (20.3-26.8)		2.39 (1.27-4.50)	0.007	2.00 (0.93-4.30)	0.077
Cytokines			0.149				
No	24	19.5 (15.1-23.9)		1.00 (referent)		1.00 (referent)	
Yes	41	25.4 (21.2-29.5)		0.65 (0.35-1.20)	0.169	0.55 (0.28-1.10)	0.089

## Discussion

HCC is a malignant tumor with poor prognosis (1). About 80% of patients have multiple metastases at the time of diagnosis and lose the chance of surgery. Even for the patients with early liver cancer who are eligible for surgery, the probability of recurrence after surgery is more than 50% (6). In terms of the treatment of HCC, surgery and radiofrequency are the main treatment regimens for early-stage liver cancer, while the intrahepatic lesions in the middle and late stages are mainly controlled by intervention. However, local control after TACE is inferior to the curative treatment. In a study of TACE involving 265 HCC patients, the 3-year local control rate is 41% and 9% for those who have tumors with complete and incomplete lipiodol accumulation, respectively (7). Radiotherapy can make up for the failure after the intervention and improve the local control rate of the disease.

Beside those patients, for patients with postoperative recurrence, even isolated liver lesions are often unable to undergo operation or radiofrequency due to insufficient residual liver volume. Meanwhile, isolated recurrent and metastatic lesions of lung, lymph node, bone and soft tissue mass have no chance of surgery. In such situation, radiotherapy can play a quite well role in disease control for those patients.

In the current study, we studied the HCC patients classified as Child-Pugh grade A or B who were unsuitable candidates for resection or radiofrequency ablation or with residual disease after TACE, and 5Gy*10f hypofractionated radiotherapy based on TOMO was given. The results showed that this segmentation and dose could achieve excellent tumor local control. At least after 2 years, none of the patients showed local recurrence of radiotherapy. To be safe, we explored some necessary parameters and indicators, such as the dose of the average liver controlled below 22Gy, and the volume was guaranteed to be above 700 mL (4). Additionally, the specially protected liver was intentionally circled, the average dose was controlled below 8Gy, and the volume was guaranteed to be above 400 mL. Therefore, all of our 65 patients were well tolerated, and none of them stopped treatment due to any gastrointestinal adverse reactions. There were no serious abnormalities in liver function and routine blood labs.

With increasingly accurate and effective local treatment of HCC, it is vital to delay the recurrence or control the distant metastasis through effective systemic treatment. From the patients we enrolled, the patients were mainly in the late stage or had failure after intervention. Therefore, although satisfactory local control rate was achieved through radiotherapy, the survival time of the patients was affected by the progression of the lesions besides the radiotherapy. Whether combination or continuous systemic treatment is required during radiotherapy or whether sorafenib can be used as an antiangiogenic targeted drug or an immune agent remains to be further explored. Recently, there are more and more reports on the immunological synergistic effect of large fraction radiotherapy in combination with immunotherapy. It has been reported in both NATURE (8) and CA Cancer (9) that this synergistic effect of radiotherapy and immunity is considered because 1) radiotherapy leads to the release of neoantigen; 2) radiotherapy changes the immune microenvironment and turns cold tumor into hot tumor; and 3) radiotherapy induces local T-Cell chemotaxis. In our current study, some patients who received cytokine treatment (GM-CSF/IL-2) (10) in combination with radiotherapy had better prognosis than those who did not receive such regimen. Similarly, after disease progression, patients who used PD-1 antibody had an advantage in survival time. Of course, our sample size is relatively small and needs further verification.

In conclusion, our study preliminarily confirmed that hypofractionated radiotherapy (5Gy*10f) based on TOMO achieved high local control rate and satisfactory OS, and such regimen was safe and well tolerated. Collectively, our findings provided valuable insights into clinical references for advanced or recurrent HCC.

## Data Availability Statement

The original contributions presented in the study are included in the article/supplementary material. Further inquiries can be directed to the corresponding author.

## Ethics Statement

The study was reviewed and approved by ethics committee of Comprehensive Cancer Center of Drum Tower Hospital of Nanjing University. The patients/participants provided their written informed consent to participate in this study.

## Author Contributions

JS, JY, and BL conceived and designed the experiments. JS, JY, SZ, WK, and JL performed the experiments and analyzed the samples. JS, SHZ, WWK, ZYZ and SSL analyzed the data. JS and BL wrote the manuscript. All authors contributed to the article and approved the submitted version.

## Funding

This study was supported by National Natural Science Foundation of China (No. 81401969); Jiangsu Provincial Medical Youth Talent (No. QNRC2016043); and the Key Medical Science and Technology Development Project of Nanjing (No. ZKX16032).

## Conflict of Interest

The authors declare that the research was conducted in the absence of any commercial or financial relationships that could be construed as a potential conflict of interest.
